# Game of neutrophils: modeling the balance between apoptosis and necrosis

**DOI:** 10.1186/s12859-019-3044-6

**Published:** 2019-12-10

**Authors:** Alva Presbitero, Emiliano Mancini, Filippo Castiglione, Valeria V. Krzhizhanovskaya, Rick Quax

**Affiliations:** 1 ITMO University, Saint Petersburg, Russian Federation; 2 University of Amsterdam, Amsterdam, the Netherlands; 3 IAC- National Research Council of Italy, Rome, Italy

**Keywords:** Neutrophils, Evolutionary game theory, Apoptosis, Necrosis, Mean-field approximation, Cellular automata

## Abstract

**Background:**

Neutrophils are one of the key players in the human innate immune system (HIIS). In the event of an insult where the body is exposed to inflammation triggering moieties (ITMs), neutrophils are mobilized towards the site of insult and antagonize the inflammation. If the inflammation is cleared, neutrophils go into a programmed death called *apoptosis*. However, if the insult is intense or persistent, neutrophils take on a violent death pathway called *necrosis*, which involves the rupture of their cytoplasmic content into the surrounding tissue that causes local tissue damage, thus further aggravating inflammation. This seemingly paradoxical phenomenon fuels the inflammatory process by triggering the recruitment of additional neutrophils to the site of inflammation, aimed to contribute to the complete neutralization of severe inflammation. This delicate balance between the cost and benefit of the neutrophils’ choice of death pathway has been optimized during the evolution of the innate immune system. The goal of our work is to understand how the tradeoff between the cost and benefit of the different death pathways of neutrophils, in response to various levels of insults, has been optimized over evolutionary time by using the concepts of evolutionary game theory.

**Results:**

We show that by using evolutionary game theory, we are able to formulate a game that predicts the percentage of necrosis and apoptosis when exposed to various levels of insults.

**Conclusion:**

By adopting an evolutionary perspective, we identify the driving mechanisms leading to the delicate balance between apoptosis and necrosis in neutrophils’ cell death in response to different insults. Using our simple model, we verify that indeed, the global cost of remaining ITMs is the driving mechanism that reproduces the percentage of necrosis and apoptosis observed in data and neutrophils need sufficient information of the overall inflammation to be able to pick a death pathway that presumably increases the survival of the organism.

## Background

Neutrophils play a crucial role in the human innate immune response. These human immune cells are not only the most abundant among immune cells, but also are the first to arrive at the sites of insult, where they execute their anti-inflammatory functions [1–3]. Approximately 10^11^ neutrophils circulating in the bloodstream [4] undergo programmed cell death, also known as *apoptosis,* per day. Neutrophils still undergo this natural death process even under healthy conditions in order to maintain homeostasis [5, 6]. In case of an insult, inflammation triggering moieties (ITMs) like bacterial lipopolysaccharides (LPS) and extracellular nucleotides induce inflammation, an essential mechanism of the innate immune response [7]. These ITMs are phagocytosed by active neutrophils or neutralized through the release of granules, depending on the nature of the ITMs. When inflammation is easily resolvable by the available population of immune cells at the site of inflammation, previously active neutrophils proceed to apoptosis [8]. However, if the inflammation is too intense or persistent, such as in systemic inflammation where ITMs are simultaneously originating from various sources in the body, neutrophils take on a dangerous death pathway called *necrosis*. Necrosis is a violent cell death that involves the rupture of the cell membrane, spilling out its cytoplasmic contents into the site of inflammation, thus further aggravating the inflammation. Necrosis is one of the major causes of tissue damage during inflammation, contributing to sepsis and potentially leading to lethal complications during the inflammation. It has been observed that the proportion of neutrophils going into either death pathway depends on the scale of the insult [9, 10]. The intricate details and the underlying mechanisms regarding the resolution of inflammation by neutrophils is still an active evolving field. For a comprehensive review, see [11].

It is reasonable to assume that the tradeoff between the evolutionary cost and benefit of inflammation, more specifically the choice of death pathway for neutrophils in response to various levels of insults, is the result of an optimization performed by evolution during the development of the human immune system. We refer to the cost and benefit as the overall factors that affect the reproduction rate and the energetic cost of the organism.

In the modern era where availability of medical treatment has paralleled the surge of technology, and novel breakthroughs in modern medical research and development has redefined state-of-the-art medical care [12], the human physiology, however, is not optimized with respect to the modern environment because evolution happens on a much larger timescale than a few centuries. Consequently, the body’s tendency to “*overreact*” to pathogens in the form of inflammation might be unnecessarily large in terms of intensity when faced with the present environment as compared to the environment millennia ago, when healthcare was not yet developed. It has been hypothesized in a seminal work by Okin et al. that given the high-cost feature of the human inflammatory response, a suboptimal tradeoff between the cost and benefit of inflammation has a high probability to adversely affect the fitness thus causing disease [13].

In the present article we extend the concepts presented in [14] by introducing the use of evolutionary game theory to model the choice of death pathways in neutrophils given an initial concentration of inflammation triggering moieties (ITMs). To the best of our knowledge, this is the first time that a subsystem of the innate immune response is modeled in the context of evolutionary game theory. Existing models found in literature use ordinary and partial differential equations [15–17], as well as agent-based and cellular automata models [18–20]. In the previous article [14], we limited our study to the use of mean-field of the interactions between neutrophils. Here, we introduce also the cellular automata model to better detail these interactions and to conform to the realistic limitation of the propagation of stimuli due to the physical constraints of biological tissues.

For the sake of simplicity, we will assume the human body to be an ensemble of biological (cellular) components directly or indirectly interacting and influencing each other. These cellular components are neutrophils that could either go into *apoptosis* or *necrosis* when responding to an insult in a specific body tissue. Since the choice of pathway of one neutrophil could influence that of another, we utilize the concept of game theory in which components of the system can “play” among them and where their interactions determine the dynamics of the system. Game theory is a branch of mathematics dealing with interactions between “rational decision makers”, called *players* (neutrophils in tissue in our case), where each player can perform one of several actions called *strategies* (here, apoptosis or necrosis), based on well-defined preferences among various outcomes represented by numerical *payoffs*.

The classical interpretation of game theory is that the analyzed *game* (i.e. the game of neutrophils) can be played exactly once by fully rational individuals who know the details of the game, which include the outcomes of each other’s strategies. *Evolutionary game theory* (EGT), on the other hand, considers the game that is played repeatedly over a long period of time by living entities that are “pre-programmed” to choose a certain strategy [21]. In the context of this study, a living entity can therefore only choose a single strategy in a single lifetime and the game is played by different generations of players for long periods of time. In essence, evolutionary game theory combines evolutionary ecology with game theory.

The benefit of apoptosis is apparent, since apart from getting rid of a small amount of ITMs, it also triggers macrophages to secrete anti-inflammatory mediators like anti-inflammatory cytokines, which lead to the resolution of inflammation [8]. In contrast, necrosis carries a risk to the body as it triggers death of a cell that damages the surrounding tissue, further aggravating inflammation. Yet on the other hand, necrosis indirectly triggers potentially life-saving actions such as the recruitment of more neutrophils into the site of inflammation by releasing pro-inflammatory mediators called pro-inflammatory cytokines. In other words, necrosis sacrifices a “local” cost to the tissue while simultaneously leading eventually to a “global” benefit to the organism (surviving and reproducing).

We define necrotic neutrophils in tissue (*necrotic entities),* in the context of game theory, as *cooperators* or *altruists* because these players are willing to undergo a violent death as well as inflict local tissue damage for the greater good. To conform with the EGT common terminology, we further define apoptotic neutrophils in the tissue (*apoptotic entities)* as *defectors* or *cheaters* for the reason that these *cheaters entities* in fact acquire a number of benefits due to the “sacrifice” made by the altruistic *necrotic entities*. Given the inherently stochastic nature of biological processes, we formulate the “game of neutrophils” as a mixed strategy game: players could choose a strategy with a certain probability, which is interpreted as the fraction of the population choosing a set of strategies. The payoffs correspond to the evolutionary fitness, and the game dynamics acquired correspond to the stable evolutionary population dynamics observed in biological systems and as the outcome of the evolutionary game [22, 23]. We hypothesize that the scale of insult pertaining to the concentration of ITMs that remain in the system is the main force that dictates the optimized balance of apoptosis and necrosis that most likely ensures the survival of the system. By using evolutionary game theory, we are able to verify that the global cost of remaining ITMs is the driving mechanism that describes the balance between the two death pathways for neutrophils given the level of insult as well as interpret on a molecular level how this balance is achieved by coupling evolutionary game theory with cellular automata.

## Methods

### The neutrophil game

The neutrophil game is a two-player game that utilizes one of the two strategies namely: *necrosis* or *apoptosis*. The payoffs for each pair of strategies are summarized in Table 1. A row corresponds to the strategy played by player 1 and a column corresponds to the strategy played by player 2. The neutrophil game follows a mixed strategy game, meaning that we assign a probability for each strategy. This allows a player to probabilistically choose between apoptosis and necrosis. The rationale behind this choice is that the pathways leading to apoptosis or necrosis depend on a series of biochemical reactions in response to external stimuli and internal processes of the cell. All these processes are stochastic, so that a cell exposed to a given environment might take one pathway or the other with a certain probability. In our model, player 1 chooses to go into necrosis with a probability *q* and apoptosis with a probability (1 − *q*). Player 2, on the other hand, chooses to go into necrosis with a probability *p* and apoptosis with a probability 1 − *p*.

**Table 1:** Payoff Matrix of the Neutrophil Game

		Player 2	
		Necrosis (*p*)	Apoptosis (1 − *p*)
Player 1	Necrosis (*q*) *m*: ITMs neutralized by granules per necrotic neutrophil	*A*, *A*	*B*, *C*
	Apoptosis (1 − *q*) *n*: ITMs neutralized by granules per apoptotic neutrophil	*C*, *B*	*D*, *D*

The neutrophil game is a symmetric game where all players are of the same type, leading to a symmetric payoff matrix. For instance, both players 1 and 2 will receive a payoff of C if *playing Apoptosis* while the other player is *playing Necrosis*.

We specify the payoffs of the neutrophil game as follows:

1 $$ A=-{c}_{Necrosis} $$

2 $$ B=-{c}_{Necrosis}+{b}_{Apoptosis} $$

3 $$ C={b}_{Apoptosis} $$

4 $$ D=2{b}_{Apoptosis} $$

When a necrotic entity plays the game with another necrotic entity, they both pay the cost *c*_*Necrosis*_, which biologically corresponds to the local tissue damage caused by the spilling out of the neutrophil’s cytoplasm contents into the surrounding tissue. A necrotic entity playing with an apoptotic entity pays a cost ***c***_***Necrosis***_ corresponding to the tissue damage and receives the benefit of apoptosis *b*_*Apoptosis*_ corresponding to the anti-inflammatory effects of apoptosis during inflammation. It also follows that an apoptotic entity playing with another apoptotic entity receives 2*b*_*Apoptosis*_.

**Fig. 1:**
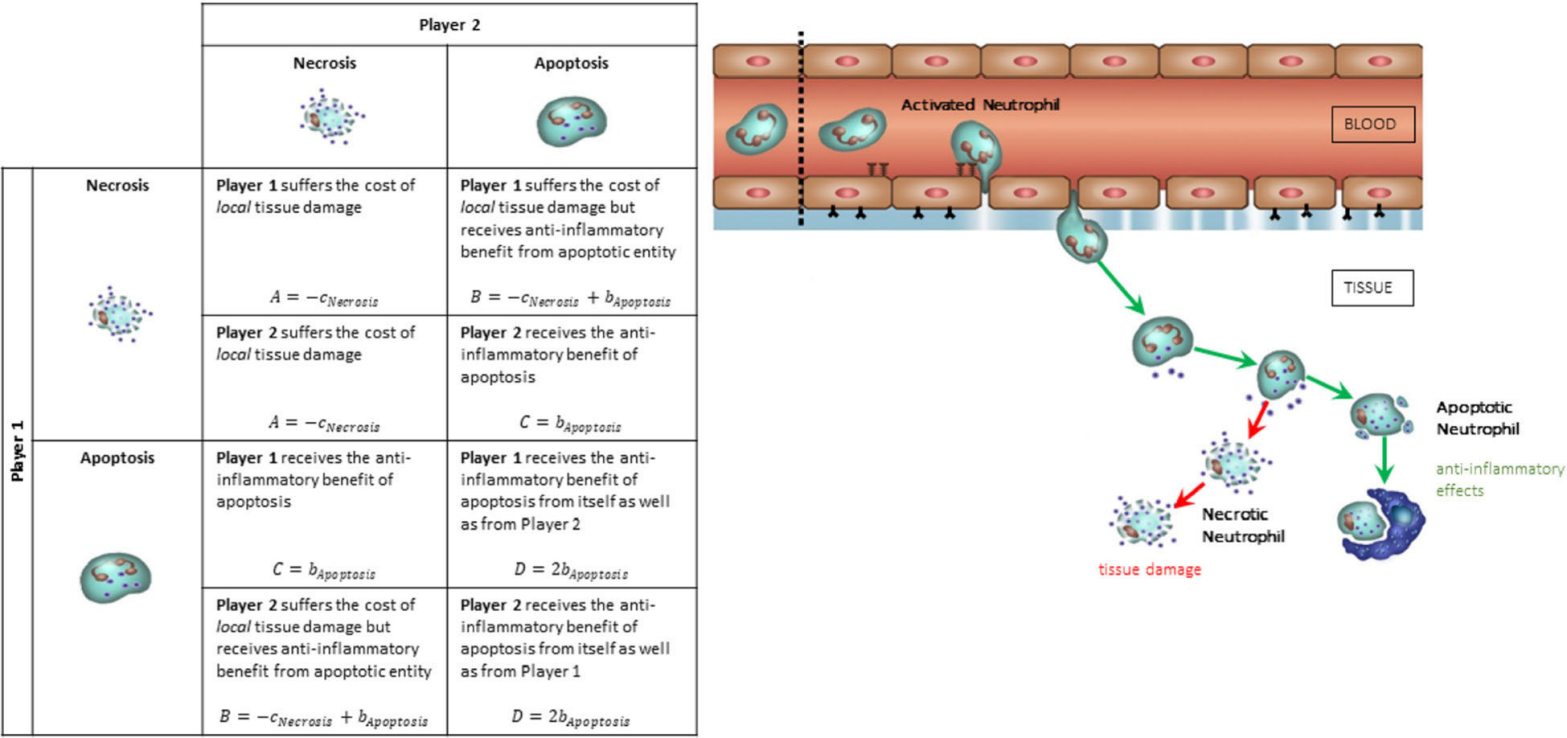
Biological Mechanism to Evolutionary Game Theory Mapping. In the case of an insult or presence of inflammation triggering moieties in the tissue, neutrophils circulating the bloodstream enter the tissue through the endothelial barrier. After the inflammation is fully neutralized by the neutrophils, they go into a programmed death called *apoptosis*, which has anti-inflammatory effects on the system. However, if the insult is too intense or persistent, the neutrophils take on a violent death pathway called necrosis that involves spilling out all of its cytoplasmic contents into the surrounding tissue, causing local tissue damage and further aggravating inflammation. The table on the left summarizes the payoff matrix of the game that the neutrophils play. In the event that both players play necrosis, each of them suffers a cost of ***c***_***Necrosis***_, pertaining to the local tissue damage each one endures. However, in the case when both players play apoptosis, each one of them receives the benefit of apoptosis from itself as well as from the neutrophil it is playing with, garnering a payoff to a total of **2*b***_***Apoptosis***_

An overview of the biological mechanism and its mapping to evolutionary game theory is summarized in Fig. 1.

### Mean-field approximation

Let *F*(*q*, *p*) be the average payoff to an individual playing necrotic “ ” of the time in a population which overall plays necrotic “ *p* ” of the time.

5 $$ F\left(q,p\right)= qpA+q\left(1-p\right)B+\left(1-q\right) pC+\left(1-q\right)\left(1-p\right)D-{e}^{{\alpha ITMs}_{remaining}} $$

The last term in (5) models the cost of the remaining ITMs in the system. An exponential is chosen to ensure that the last term in (5) does not become negative. In addition, by using this exponential term, we were able to match our model against the data as shown in the Results and Discussion (Data Parameter Space), as well as being amenable to analytical simplification. However, we do suspect that sub-linear forms such as logarithms would not work since it would not necessarily outweigh the linearly increasing positive pay-off contributions. We assume that the term *αITMs*_*remaining*_ in Eq. (5) corresponds to the global cost of the individual playing necrotic with probability *q* due to the “threat” of remaining ITMs in the system, where *α* is the “strength” of this global cost that we have calibrated against the data presented in Section 0. *ITMs*_*remaining*_ is defined as follows:

6 $$ {ITMs}_{remaining}={ITMs_{initial}}^k- pm\left({N}_T-1\right)- qm-\left(1-p\right)n\left({N}_T-1\right)-\left(1-q\right)n $$

where *ITMs*_*initial*_ corresponds to the initial concentration of ITMs, *N*_*T*_ is the total number of neutrophils, *n* corresponds to the amount of ITMs a single apoptotic neutrophil could neutralize while *m* is the amount of ITMs a single necrotic neutrophil eventually neutralizes through the recruitment of additional neutrophils, and *k* models the observed power law behavior in the data [9] of necrotic neutrophil population with respect to the initial concentration of ITMs. See Section 0 for a more detailed explanation of how *k* was obtained.

John Forbes Nash demonstrated that there is always at least one mixed strategy equilibrium in a finite game (i.e., having a finite number of strategies, in our case two) [24]. In order to find this equilibrium, we calculate the optimal strategy of a player choosing necrosis with probability *q* by taking the partial derivative of Eq. (5) with respect to *q* and equating it to zero as shown in Eq. (7).

7 $$ \frac{dF\left(q,p\right)}{\delta q}= pA+\left(1-p\right)B- pC-\left(1-p\right)D-\alpha \left(n-m\right){e}^{\alpha \left[{ITMs_{initial}}^k- pm\left({N}_T-1\right)- qm-\left(1-p\right)n\left({N}_T-1\right)-\left(1-q\right)n\right]}=0 $$

Because *F*(*q*, *p*) = *F*(*p*, *p*) when the optimal *q*^∗^ = *p*, it is clear to see that *F*(*q*, *p*) reaches a *maximum* at *q*^∗^ = *p*, provided that 0 < *p* < 1. Hence, the optimal *q*^∗^ must be equal to *p* in the stationary state. In fact, it would be internally inconsistent if each neutrophil decides with probability *q* while at the same time the population average remains probability *p* ≠ *q*.

The optimal value *p* = *q*^∗^ represents the optimal population of necrotic neutrophils given the values of payoff *b*_*Apoptosis*_, *c*_*Necrosis*_, global cost factor *α*, neutralizing factors *m* and *n*, total neutrophils *N*_*T*_, and *ITMs*_*initial*_.

8 $$ p={q}^{\ast }=-\frac{\ln \left(\frac{b_{Apoptosis}+{c}_{Necrosis}}{\alpha \left(m-n\right)}\right)+\alpha n{N}_T-\alpha {ITMs_{initial}}^k\ }{\alpha m{N}_T-\alpha n{N}_T} $$

with the following mathematical constraints:

9 $$ {\displaystyle \begin{array}{c}{b}_{Apoptosis}+{c}_{Necrosis}\ne 0\\ {}m\ne n\\ {}\alpha \ne 0\end{array}} $$

In conclusion, we can define the average fitness ***F***(***q***, ***p***) of necrosis by using the payoffs specified in Table 1 together with the global cost of the remaining ITMs. In our model we optimize this average fitness during evolutionary time. The game is played via mean-field interactions between players represented by the average evolutionary fitness in Eq. (5). The game ends when an evolutionary stable point is achieved as specified in Eq. (7). This means that the percentage of necrotic population does not change anymore through evolutionary time. This steady state is reached at the value of necrotic *p* calculated in Eq. (8). The outcome of this evolutionary game corresponds to the stable population dynamics, which we can then compare to that of biological systems.

### Cellular automata

What we have described so far is a mean-field type of interaction, where neutrophils interact with equal probability with *all* the other neutrophils in the system. In other words, each neutrophil “*plays*” the game with *the ensemble of all* other neutrophils. However, in biological systems these interactions among neutrophils (either direct or indirect through the release of cytokines and other stimuli) are spatially limited by the characteristic lengths of biological signals (such as diffusion lengths) that characterize the extent at which these stimuli are released and propagated in the body. Hence, a mean-field assumption may neglect the role of spatial interactions observed in biological systems [19], and may not in fact represent faithfully what we observe biologically in real systems.

By employing cellular automata, we aim to gain deeper insights into the microscopic interactions that occur among neutrophils when spatial proximity is taken into account [19]. We model this type of interaction as follows:

First, we create an empty lattice of size 50 × 50. In our model, the entire lattice space is interpreted to correspond to a small portion of tissue. In order to model a bigger tissue that represents the entire body, we use periodic boundary conditions in our simulations. Additionally, since we assume a systemic inflammation, where ITMs are simultaneously originating from various sources in the body, we assume that the concentration of ITMs are equally distributed all over the simulated lattice. This follows that the distribution of the neutrophils in the tissue should also be homogenous. Hence, a single lattice site is occupied by a *neutrophil entity*, which, in the context of our model, refers to the neutrophil cell and the tissue the neutrophil occupies. The 50 × 50 lattice site, therefore, contains a total of 2500 neutrophils. Because we do not have spatial information of choice of death pathway, we picked the simplest topological feature, which is a square lattice site. We assume that the exact local spatial structure is not important, as long as the locality of the interactions between neighboring neutrophils in the tissue is reproduced in order to allow for local fluctuations.

Biologically, 2500 neutrophils occupy a volume of 1 *mm*^3^ in the blood [25]. However, this volume is increased by a factor of 15, as the neutrophils diffuse from the bloodstream, a smaller “chamber” with volume approximately equal to 5 l, into the tissue, which is a much larger “chamber” of 75 l, assuming an 80 kg person. One could then imagine that these 2500 neutrophils will be occupying a volume of 15 *mm*^3^ in the tissue, where a single neutrophil occupies a 6 *μm*^3^ and the distance from one neutrophil to the other is 182 *μm*. Our model is a simplification of the dynamics of neutrophils in two dimensions, due in part to the limitation of spatial information in the data. Hence, our lattice site that contains 2500 neutrophils corresponds to a 82.5 *mm*^2^ patch of tissue. Neutrophils are able to “communicate” with each other via the spillage of cytoplasmic content or the release of chemical signals such as cytokines. The distance of one lattice site to the adjacent site corresponds to 182 *μm*, which is a value that is in fact well within the communication range of neutrophils via messenger proteins called cytokines [26]. Hence, a neutrophil entity is well able to communicate with its nearest neighbors. We simplify this scenario in our model by putting the neutrophil entities next to each other in the lattice. Additionally, this reduced the processing time, as the range of the Moore neighborhood is simply a lattice site away.

ITMs are introduced homogenously by distributing the total ITM concentration equally in the lattice. In the simulation, this is simply calculated by assigning the concentration of ITMs to a global value that is neutralized at every time step.

The algorithm commences by choosing an activated neutrophil randomly inside the 50 × 50 lattice that is initially filled with 2500 activated neutrophils. The chosen activated neutrophil plays the game based on the payoffs summarized in Table 1 with all of its immediate neighbors in a Moore neighborhood of range equal to 1 [27].

The choice of strategy of an activated neutrophil is decided based on a fitness comparison made by choosing either strategy. The payoff the activated neutrophil gets from playing with *all* its immediate neighbors is reduced by the cost of the remaining ITMs in the system. We emphasize that the fitness calculated in cellular automata is similar to the fitness we calculated in the mean-field scheme: the fitness for picking a strategy is reduced by the cost or “threat” of the presence of ITMs that remain in the system. This total payoff for apoptosis or necrosis is compared, and the strategy with the greater payoff is chosen as the winning strategy for the current iteration.

When the payoff for going into apoptosis is exactly the same as deciding on necrosis, the activated neutrophil takes the apoptotic pathway. We model it this way because we assume that apoptosis is the default pathway that neutrophils take with or without inflammation. It is after all a type of programmed death that take place regardless of the intensity of inflammation.

The process for a single activated neutrophil choosing a strategy corresponds to a single iteration or time step in the algorithm. The same steps are done to all the activated neutrophils in the lattice. Hence, the algorithm stops when all activated neutrophils have picked a strategy. The outcome is a percentage of necrosis as well as apoptosis given the initial concentration of ITMs.

What is known so far is that the more intense the inflammation is, the greater the percentage of necrosis. This means that in one way or another, neutrophils have a way of “sensing” any presence of threat in the system either directly from the concentration of ITMs nearby or indirectly via release of chemicals from other neutrophils and immune cells. Mechanisms regarding the recruitment of neutrophils into the site of inflammation have been well-documented and is still largely an active field [28–30]. However, the extent to which an activated neutrophil “senses” this threat, remains relatively unknown. What is known is that neutrophils are directed towards an inflammation via the release of pro-inflammatory cytokines. Pro-inflammatory cytokines are produced by macrophages upon engulfment of ITMs at the site of inflammation. Pro-inflammatory cytokines are also produced when necrotic neutrophils neutralize ITMs. On the other hand, anti-inflammatory cytokines, which controls the response of pro-inflammatory cytokines, are secreted by macrophages upon phagocytosis of apoptotic neutrophils. Hence, we introduce two hypothetical mechanisms for calculating fitness, which will hopefully shed light to the biological mechanisms: *Local ITMs Scheme* and *Global ITMs Scheme*.

***Local ITMs Scheme*** explores the assumption that activated neutrophils choose to go into apoptosis or necrosis based on two conditions: 1) intensity of *local* inflammation – pertaining to the concentration of ITMs that are in its proximity and 2) strategies of its immediate neighbors, which considers the summation of strategies within the neighborhood much like a majority rule. This condition is based on the cumulative effect of the release of stimulators, such as pro- and anti-inflammatory cytokines by apoptotic and necrotic neutrophils that are located within the immediate neighborhood of the activated neutrophil.

***Global ITMs Scheme***, is motivated by the assumption, alternative to the one used in the local ITMs Scheme, that activated neutrophils “sense” the overall intensity of the ITMs as a result of faster diffusion of cytokines that would provide information about the overall state of inflammation in the tissue. In this scheme, activated neutrophils pick a strategy based on two conditions: 1) the cost of remaining ITMs is calculated for the entire lattice space and 2) the cumulative strategies of its immediate neighbors. This is under the assumption that the release of neutrophil by either apoptotic or necrotic neutrophils are limited only within the immediate neighborhood where the activated neutrophil is located.

We emphasize that condition 2 is the same for both local and global schemes. We claim that sensing ITMs at a long distance is mediated by the diffusion of cytokines. On the other hand, sensing the strategy of another neutrophil in the system is related to the spill of cytoplasmic content which we assume to diffuse at a slower speed. Hence, we only look into immediate neighbors surrounding the activated neutrophil and assume that cytokines diffuse faster than the cytoplasmic spill.

**Fig. 2:**
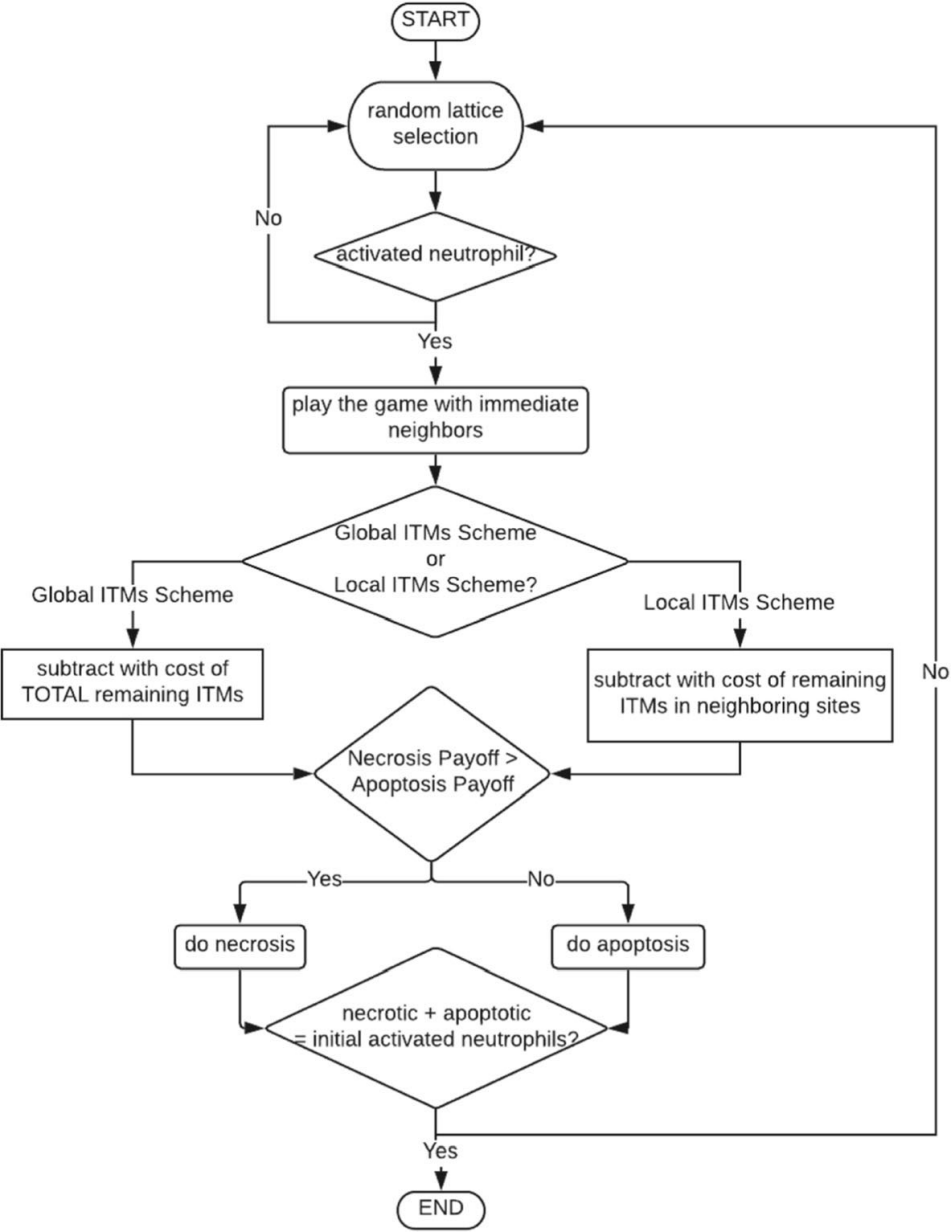
Summary of the Cellular Automata Algorithm. The iteration starts by randomly choosing a single activated neutrophil in the lattice. The chosen activated neutrophil then plays the game with all its immediate neighbors by calculating a payoff that is based on the payoff matrix specified in Table 1. The cost of remaining ITMs, on the other hand is calculated depending on the ITMs scheme (local or global) selected. The strategy with the higher payoff wins the game and is therefore chosen for this iteration. The absence of an activated neutrophil in the lattice means the end of the algorithm. The final number of apoptotic and necrotic neutrophils are counted

The summary of the cellular automata algorithm used is summarized in Fig. 2. The algorithm starts with a random lattice selection that is done on a lattice initially occupied by activated neutrophils. If the selected neutrophil is an activated neutrophil (and not an apoptotic or necrotic neutrophil, which shall be present in the succeeding runs) the chosen activated neutrophil plays the game with its immediate neighbors. Depending on whether the ITMs scheme is global or local, the cost of remaining ITMs is consequently calculated. If the payoff for necrosis is greater than the payoff for apoptosis, the activated neutrophil goes into necrosis. Otherwise, it goes into apoptosis. The choice for apoptosis or necrosis corresponds to a single iteration in the cellular automata algorithm. A checkpoint is then established (see diamond with text “necrotic + apoptotic = initial activated neutrophils?”), which counts the total number of apoptotic and necrotic neutrophils. If this value is equal to the initial number of activated neutrophils, implying that all activated neutrophils have gone into either apoptosis or necrosis, the algorithm ends. Otherwise, a random lattice selection is made again until the checkpoint condition is satisfied.

### Code implementation and repository

We used Python 3.7 on a 3.30 GHz Intel® Core™ i7-5820K CPU with 16.0 GB RAM in all our simulations. The code has been uploaded to https://github.com/avpresbitero/GON.

## Results and discussion

### Model results for a changing environment

In this section, we take a closer look into how the mechanisms we have identified (*α*, *b*_*Apoptosis*_, *c*_*Necrosis*_, *m* and *n*) contribute to the final distribution of necrotic and apoptotic strategies in the system. In order to do this, we explore how nature plays the evolutionary game to arrive at optimal fractions of neutrophil population when certain parameters are varied. The motivation for this is that certain factors that affect the cost and benefit of choosing either strategy is assumed dependent on the type of environment the human body is exposed to. We have identified the following parameters *α*, *b*_*Apoptosis*_, *c*_*Necrosis*_, *m* and *n* as determining factors to the fitness of neutrophils. However, due to a high degree of freedom that our model exhibits, a global sensitivity analysis would not deliver meaningful results. Hence, we vary the parameters one at a time for different values of *m* and look at the effect it has on the stable distribution of strategies in the system.

**Fig. 3:**
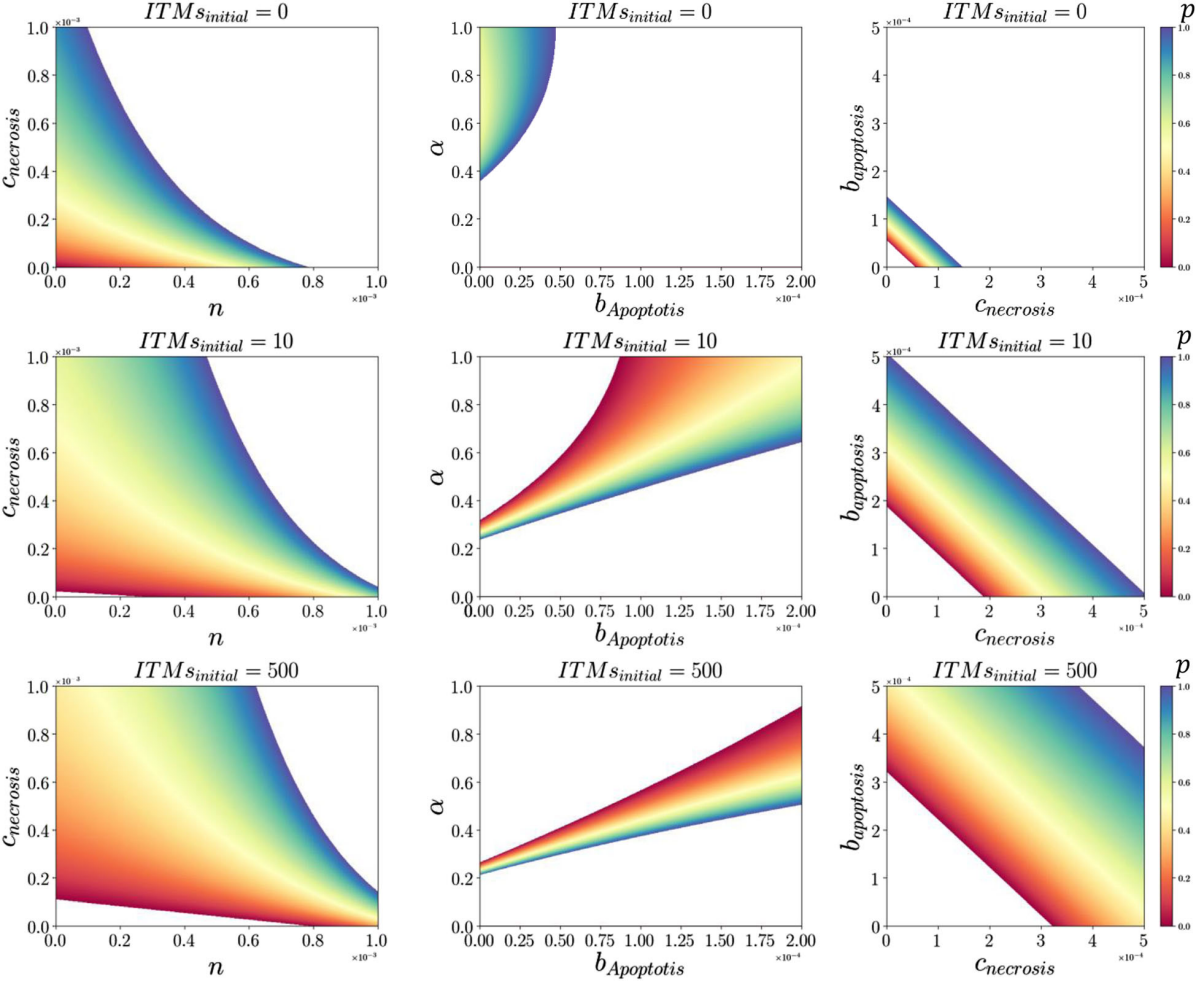
Exploring the payoff matrix for (left column) ***c***_***Necrosis***_ and ***n***, (middle column) ***α*** and ***b***_***Apoptosis***_ and *(right column)* ***b***_***Apoptosis***_ and ***c***_***Necrosis***_ with varying ***ITMs***_***initial***_. The colors correspond to the evolutionary stable fraction of necrotic neutrophils after the game is played for varying concentrations of initial insult. A system that stabilizes at necrosis is shown dominantly red. Conversely, a system that stabilizes at apoptosis is shown in blue. Note that black area denotes solutions that are invalid. This includes *p* (fraction of necrotic neutrophils) that do not lie within the interval [0, 1], and those that do not obey the constraints set in Eq. (9). *N*_*T*_ = 2500 was held constant for all simulations. On the *left column*, we set the value *α* = 1.0, *b*_*Apoptosis*_ = 0.0001, and *m* = 0.0015. In the *middle column*, *n* = 0.0004, *m* = 0.0008, *c*_*Necrosis*_ = 0.0001 were held constant. In the *right column*, *α* = 1.0, *n* = 0.0004, and *m* = 0.0008 were fixed. The black area corresponds to invalid solutions

We start by looking at the optimal fractions of necrotic populations (*p*) by simultaneously varying the cost of necrosis (*c*_*Necrosis*_) and the amount of ITMs a single apoptotic neutrophil can neutralize (*n*) in Fig. 3 (*left column*) while fixing parameters *α*, *b*_*Apoptosis*_, and *m*. Using our model, we show that the system evolves to an optimal fraction of necrotic and apoptotic neutrophils that corresponds to an increasing cost of necrosis as well as increasing capacity of ITM neutralization by apoptotic neutrophils as the insult intensifies. For the system to survive increasing threat due to ITMs, the system to increase its ITM resolution capacity by increasing *n* while also making it costly to go into necrosis. Necrosis, in the biological point of view, has detrimental effects to the system brought about by aggravating the initial level of inflammation by generating more ITMs.

Next we vary the global cost (*α*) and benefit of apoptosis (*b*_*Apoptosis*_) while fixing *c*_*Necrosis*_, *n*, and *m* as shown in Fig. 3 (*middle column*). Here we show that the system copes with increasing levels of insult by shifting the optimized fractions of neutrophils towards decreasing global cost and increasing the benefit of apoptosis. With all other parameters held constant, that is with a fixed ITM neutralizing capacity of both apoptotic and necrotic neutrophils coupled with also a fixed cost of necrosis, nature plays an evolutionary game where the system copes with an increasing level of insult by lowering the cost imposed by the remaining ITMs and also by increasing the benefit that can be reaped from the act of apoptosis via anti-inflammatory factors.

Finally, in Fig. 3 (*right column)*, we vary *b*_*Apoptosis*_ and *c*_*Necrosis*_ while fixing the values for *n*, *m* and *α*. Here we show that with increasing intensity of insult, the optimized fractions of neutrophils are achieved after playing the game only with increasing contributions from the cost of necrosis and benefit of apoptosis. Hence, by increasing the cost to go into necrosis coupled with increasing the anti-inflammatory effects of apoptosis, the game finds stable concentrations of neutrophil populations.

There is an apparent transition of strategy from largely necrosis to apoptosis (that is, from dominantly red to blue from left to right) as *n* (left column), *b*_*Apoptosis*_ (middle column) and *c*_*Necrosis*_ (right column) increases, thus favoring apoptosis. Hence, we interpret that increasing the cost of necrosis lowers the fitness for the necrotic strategy. Therefore, the system stabilizes into apoptosis (apparent in left and right columns). Conversely, increasing the cost of remaining ITMs (*α*) in the system shifts the equilibrium to necrosis. Note that we also see the same trends when *α* is plotted with respect to *c*_*Necrosis*_, the same as what we see in the middle column. Hence, with increasing initial concentrations of ITMs, the stable evolutionary strategies are shifted towards increasing *c*_*Necrosis*_.

### Data parameter space

Following the observation in [9], it was shown in-vitro that the proportion of neutrophils going into apoptosis or necrosis actually depends on the scale of insult. We utilize data on the peak values of necrotic population at time point 24 h. We also used an additional finding by Damas et al. where they show that 500 ng/ml LPS corresponds to the fatal concentration of LPS in humans, [31]. This corresponds to 100% necrosis, where the level of inflammation becomes uncontrollable due to the growing detrimental effects of local tissue damage generated from necrosis and the lack of anti-inflammatory functions from apoptosis. We are well aware that the assumptions we make using our model is limited by the data that we have at the moment. In particular, the power-law relation may turn out to actually be a different functional relationship when more data would be collected. However, the current work aims to provide baseline research to show what the concepts behind the model can do. Although the analytical derivations would change, we believe that the main methodology and qualitative conclusions would remain unchanged. Hopefully, our work motivates other researchers to take a deeper look into these concepts as well as invites stakeholders to generate additional data.

The data in [9] reports a 17% baseline level of necrosis even in absence of ITMs. This might be due to experimental setup and other laboratory procedures of the in-vitro experiment. However, it is reasonable to assume that no necrosis occurs in-vivo in absence of ITMs). Additionally, the data is not normalized since in the experiments not all neutrophils measured were in apoptosis or necrosis. For this reason, we correct the population of necrotic neutrophils in the data by normalizing it with respect to the total percentage of apoptotic and necrotic neutrophils used in the in-vitro study. This normalization is necessary since our model does not take into account the population of activated neutrophils that have not yet gone into apoptosis or necrosis. We show that, after such normalization, the percentage of necrotic neutrophils seem to exhibit a power-law behavior with respect to the initial concentration of ITMs that trigger an inflammatory response.

**Fig. 4:**
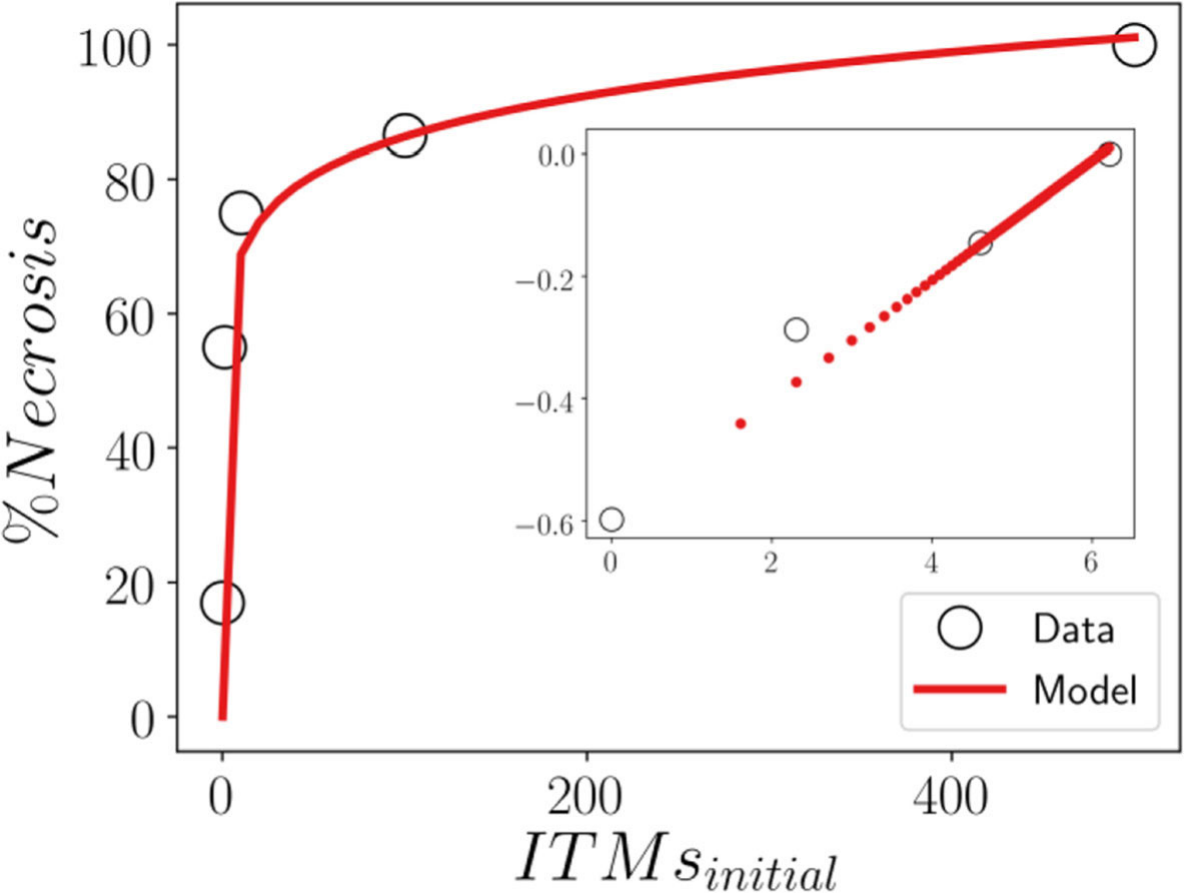
Data distribution and model output in terms of percent necrosis (*main*) and corresponding log-log plot (*inset*). Based on the log-log plot, the percentage of necrotic neutrophil population follows a power-law behavior with power *k* = 0.0929. However, at initial concentration of 10 ITMs, the data point seems to deviate from this power law behavior (*inset*), which is also seen slightly higher than the predicted percent necrosis (*main*)

We model this power-law behavior in Eq. (10):

10 $$ p-\gamma =\beta \bullet {ITMs_{initial}}^k $$

where *ITMs*_*initial*_ corresponds to the initial concentration of ITMs, *k* = 0.0929, *β* = e^−0.6^ and a constant term *γ*, which we will explain shortly. In the log-log plot we observe a linear relation between percentage of necrosis with respect to the initial concentration of ITMs. This relation has an intercept on the y axis of −0.6 and a slope k equal to 0. 0929 (Fig. 4).

However, the data report 17% necrosis even without the presence of LPS. In order to remove this baseline amount of necrosis from the experimental dataset, we subtract a constant term *γ* = 0.17 in the power-law equation. We consider this baseline necrosis as originating from sources other than a systemic inflammation, which we do not take into account in our model.

In order to determine the parameter space of the EGT model, which reproduces this experimental relation, we compare Eq. (10) with Eq. (8). It is easy to see that ***γ*** and ***β*** are as follows:

11 $$ \boldsymbol{\gamma} =-\frac{\mathbf{\log}\left(\frac{{\boldsymbol{b}}_{\boldsymbol{Apoptosis}}+{\boldsymbol{c}}_{\boldsymbol{N}\boldsymbol{ecrosis}}}{\boldsymbol{\alpha} \left(\boldsymbol{m}-\boldsymbol{n}\right)}\right)+\boldsymbol{\alpha} \boldsymbol{n}{\boldsymbol{N}}_{\boldsymbol{T}}}{\boldsymbol{\alpha} \boldsymbol{m}{\boldsymbol{N}}_{\boldsymbol{T}}-\boldsymbol{\alpha} \boldsymbol{n}{\boldsymbol{N}}_{\boldsymbol{T}}} $$

12 $$ \boldsymbol{\beta} =\frac{\mathbf{1}\ }{{\boldsymbol{N}}_{\boldsymbol{T}}\left(\boldsymbol{m}-\boldsymbol{n}\right)} $$

We can reduce this 5-dimensional parameter space (***b***_***Apoptosis***_***,c***_***Necrosis***_***,m,n,α***) to 4 dimensions by extracting ***n*** from (12), which is the equation shown in (13).

13 $$ \boldsymbol{n}=\boldsymbol{m}-\frac{\mathbf{1}\ }{{\boldsymbol{N}}_{\boldsymbol{T}}\boldsymbol{\beta}} $$

**Fig. 5:**
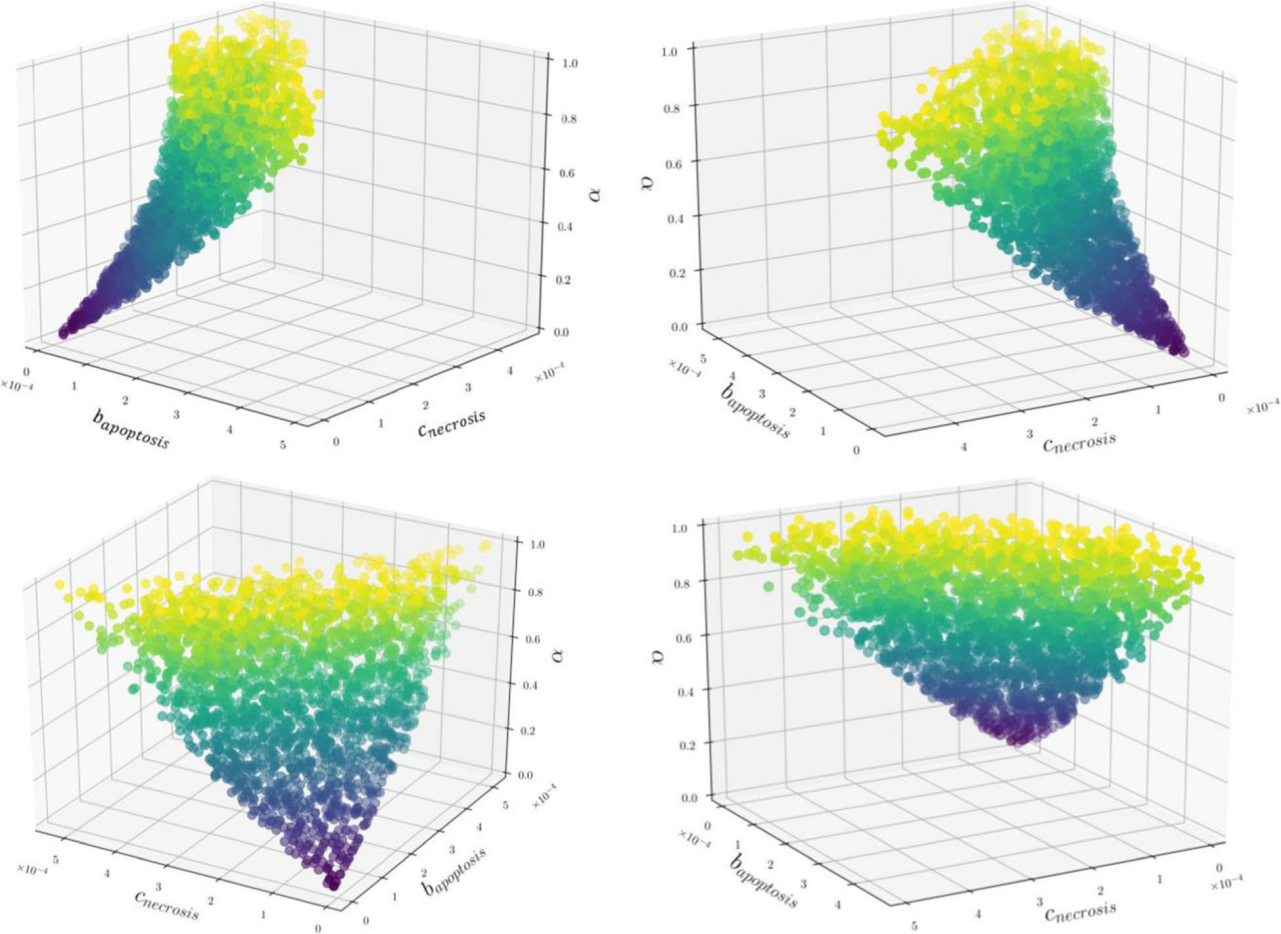
Data Parameter Space (***α***, ***b***_***Apoptosis***_, ***c***_***Necrosis***_). Each point in the plot corresponds to the combination of parameters that satisfies the data. This 3-dimensional parameter data space can be better visualized via an interactive plot, which we have uploaded to the following link: https://github.com/avpresbitero/EGTN (1). The colors are chosen only to add visual depth to the plot, where purple to yellow corresponds to increasing values of ***α***. Nature has evolved into stable percentage of necrotic and apoptotic neutrophils that correspond to a set of parameters (***α***, ***b***_***Apoptosis***_, ***c***_***Necrosis***_) that exhibit direct proportionality towards each other. That is, with higher cost of necrosis, higher values for global cost of remaining ITMs is preferred. This shows that nature made necrosis to be damaging on purpose for the organism to make sure that this event only happens a few times, that is, only when necessary

**Fig. 6:**
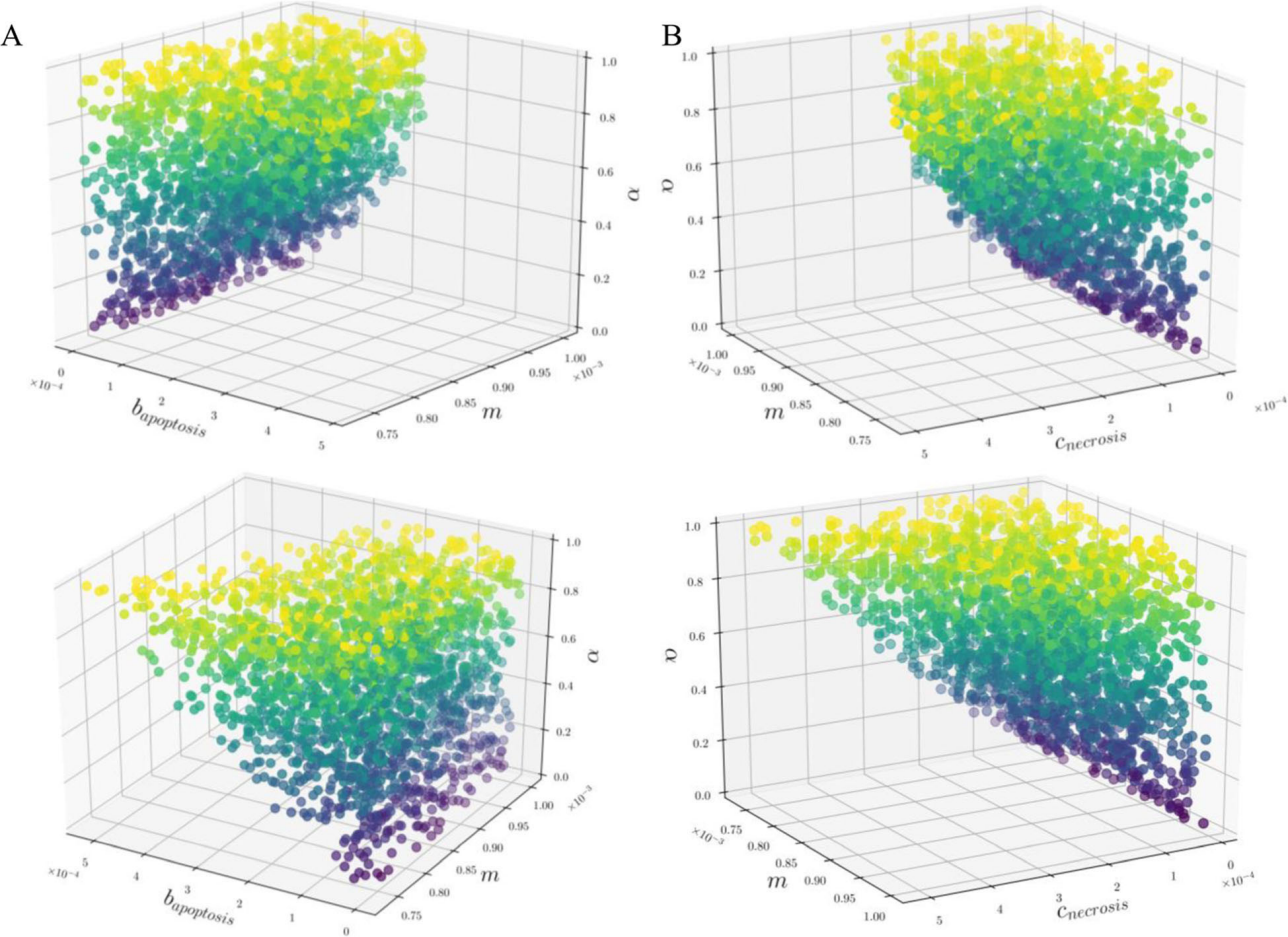
Data Parameter Space (***m***, ***b***_***Apoptosis***_, ***α***). Each point in the plot corresponds to the combination of parameters that satisfies the data. This 3-dimensional parameter data space can be better visualized via an interactive plot, which we have uploaded to the following link for (**a**) and (**b**) respectively: https://github.com/avpresbitero/EGTN (2) and (3). The colors are chosen only to add visual depth to the plot, where purple to yellow corresponds to increasing values of ***α***. The evolutionary stable percentages of neutrophils correspond to lower benefits of apoptosis and costs of necrosis when the costs of remaining ITMs become threatening to the system. Stronger resolving power of ITMs, or higher values of ***m***, correspond to narrower range and lower benefits of apoptosis and costs of necrosis in the data parameter space. Despite making necrosis detrimental to the system, nature balances the collective effect of the intrinsic property of neutrophils, cost of necrosis and benefit of apoptosis. Nature could have also made the anti-inflammatory benefits of apoptosis more effective especially when the presence of remaining ITMs becomes increasingly threatening to the organism

**Fig. 7:**
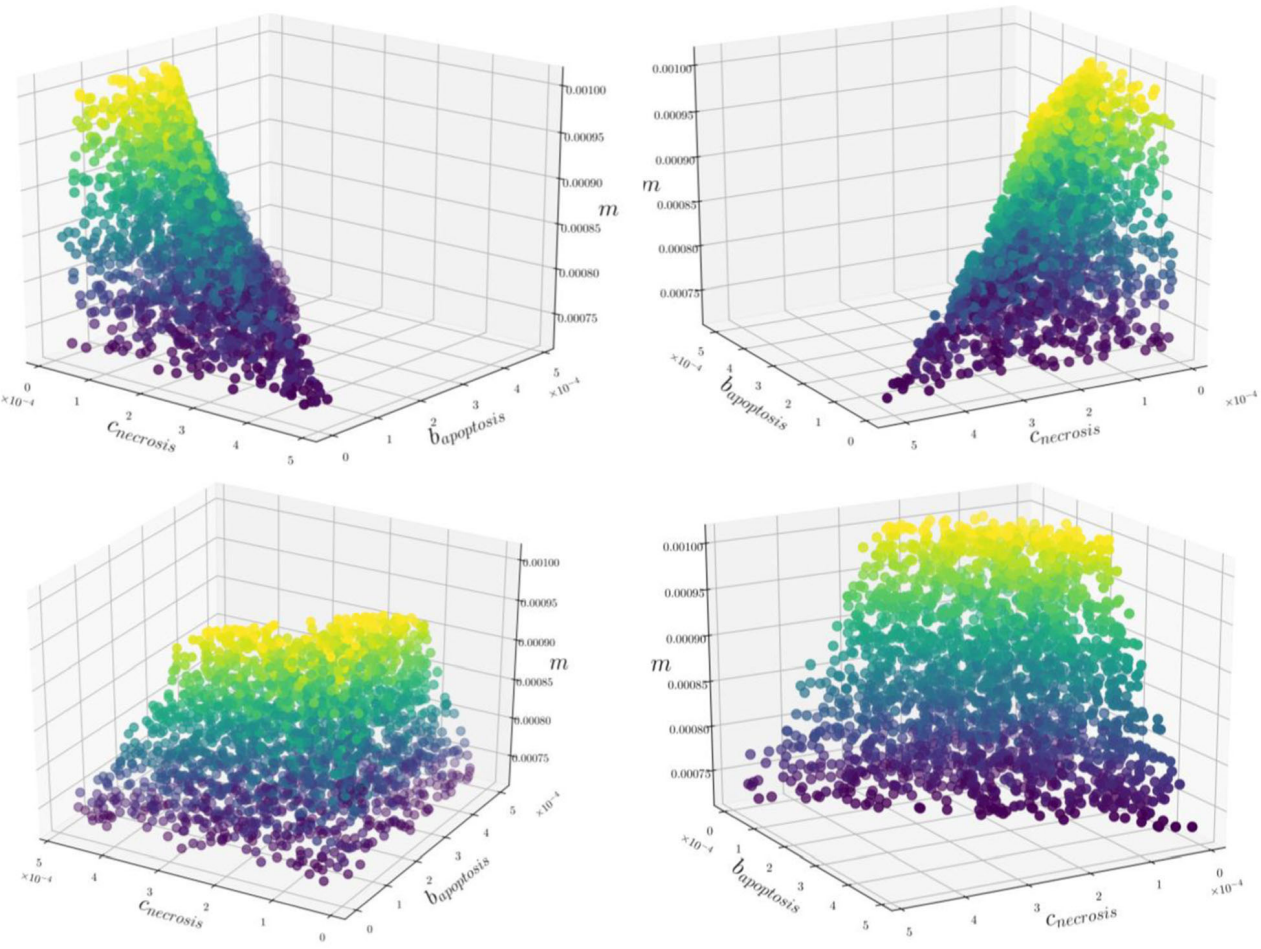
Data Parameter Space (***m***, ***b***_***Apoptosis***_, ***c***_***Necrosis***_). Each point in the plot corresponds to the combination of parameters that satisfies the data. This 3-dimensional parameter data space can be better visualized via an interactive plot, which we have uploaded to the following link: https://github.com/avpresbitero/EGTN (4). The colors are chosen only to add visual depth to the plot, where purple to yellow corresponds to increasing values of ***m***. With increasing ITM resolving strength of neutrophils, low range of values for costs of necrosis and benefits of apoptosis correspond to evolutionary stable percentages of neutrophil population. That is, with favorable intrinsic ITM resolving properties of neutrophils is compensated by lowering both costs of necrosis and benefits of apoptosis to maximize the overall fitness of the system

Based on Eqs. (11) to (13), we are able to identify the parameter space for which the relation between necrosis and ITMs reported in the dataset is fulfilled. In order to visualize the parameter space that satisfies all our conditions we plot the solutions in Figs. 5, 6 and 7.

Nature, through a course of evolutionary time, has driven neutrophils to adopt these specific set of parameter values shown in Fig. 5. We show that the system evolved into stable percentages of neutrophil population that correspond to a combination of values of ***α***, ***b***_***Apoptosis***_, ***c***_***Necrosis***_, where these three parameters have direct proportionality towards each other. That is, nature plays the evolutionary game and achieves stable percentages of neutrophil population by simultaneously opting for lower cost of going into necrosis while the global cost of remaining ITMs is also low. With higher cost of necrosis, higher values for the global cost of remaining ITMs is preferred. A higher cost for necrosis means further aggravating the current level of inflammation by inducing local tissue damage that increases the ITM levels in the system. The system, in response, imposes higher global cost of remaining ITMs in the system. This goes to show that nature might have made the necrosis death pathway purposely damaging to the organism so that the fitness will make sure that the events of necrosis are done only as few times as possible.

We visualize the same data parameter space in terms of parameters ***m***, ***b***_***Apoptosis***_, ***α*** on the left panel (A) and ***m***, ***c***_***Necrosis***_, ***α*** on the right panel (B) of Fig. 6. We show that when the threat of remaining ITMs is high, the evolutionary stable percentages of neutrophil population for high, or effective resolving capacity of neutrophils, correspond to a low range of values for benefits of apoptosis (A), and by Eq. (13), low range of values for costs of necrosis (B). This range of values for benefits of apoptosis and costs of necrosis become narrower and spans lower values when the threat of remaining ITMs is low. This is shown by the narrow wedge shape located at the bottom of the plot of panel A (shown in purple). This shows that nature could not have only made necrosis deliberately detrimental to the organism, it also could have made the anti-inflammatory benefits of apoptosis more effective especially when the presence of remaining ITMs in the system becomes increasingly threatening.

Furthermore, with stronger resolving power of ITMs, or the higher ***m*** is, the narrower the range of values for benefits of apoptosis and costs of necrosis, which spans lower values in the data parameter space. This goes to show how well the system compensates by choosing combinations of parameters that balance the intrinsic property of neutrophils to neutralize ITMs, the damaging effects of necrosis, and the anti-inflammatory benefit of apoptosis.

The preference for range of costs of necrosis and benefits of apoptosis at lower values with increasing ITM-resolving capacity ***m*** is also apparent in the data parameter space shown in Fig. 7. Nature could have made necrosis intentionally detrimental to the system, but when the intrinsic property of neutrophils to neutralize ITMs is favorable, costs for necrosis and the benefits of apoptosis adjust accordingly by having lower values. Nature could have chosen to maximize the organism’s fitness by limiting the number of neutrophils that go into apoptosis or necrosis when even a small amount of neutrophils can completely resolve the inflammation. By doing so, the organism has conserved a considerable amount of energy, which is favorable for its survival.

### Emergence of necrosis among neutrophils with limited interactions in cellular automata scheme

We use all the combinations of parameters derived in the mean-field scheme that corresponds to the experimental relation as input parameters for game played in the cellular automata algorithm. We do this under the assumption that these combinations of parameter values correspond to those that have been optimized during evolution by nature. The Global ITMs scheme assumes that an activated neutrophil bases its strategy on the strategy of its immediate neighbors and the total concentration of ITMs in the system.

**Fig. 8:**
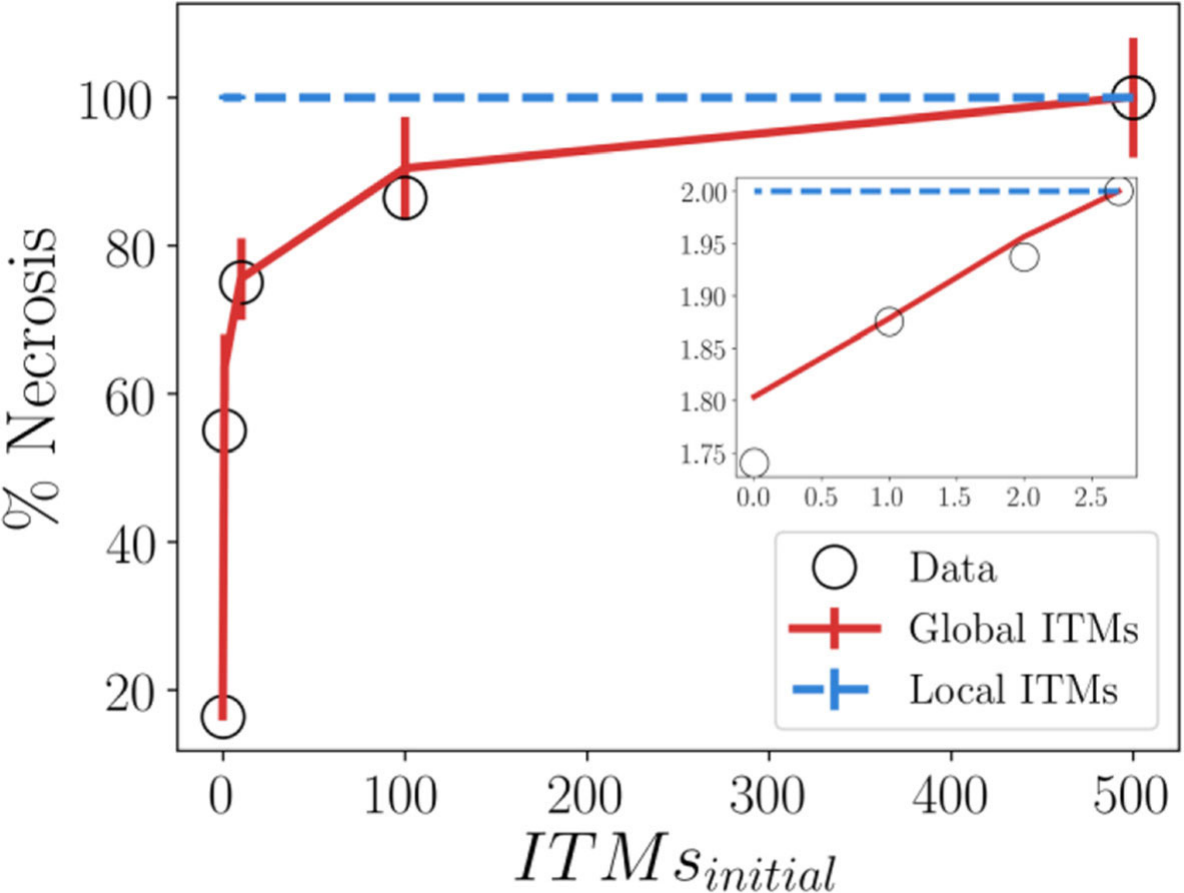
Emergence of Cooperation in Restricted Interactions via Cellular Automata (main) and Corresponding Log-Log Plot (inset). Global ITMs scheme refers to when neutrophils are able to detect the concentration of ITMs in a global scale. Local ITMs scheme, on the other hand, refers to when the neutrophils could detect the concentration of ITMs only within a certain range that is immediate to the activated neutrophil. Each point in the plot corresponds to the mean percentage of necrosis calculated from the cellular automata algorithm for all combinations of parameter values in the data parameter space given the initial concentration of ITMs. Error bars correspond to the standard deviation from the mean of percentage of necrosis calculated for all combinations of parameters in the data parameter space

Here we explore a lattice size of 50 × 50, so that the entire lattice is occupied by all 2500 neutrophils. In order to model systemic inflammation where ITMs are everywhere in the body, we 1) distribute the initial concentration of ITMs uniformly over the lattice and 2) assume periodic boundary conditions to assume a larger area of the tissue that corresponds to the entire body. Our results are summarized in Fig. 8.

The results using the Global ITMs scheme fits well with the data. On other hand, the percentage of necrosis in the Local ITMs scheme shows that regardless of initial ITM concentration, necrosis is always the dominant strategy and that this percentage of necrosis is maintained at about 100%.

The results of our simulations provide numerical evidence that indeed, neutrophils need sufficient information regarding the intensity of inflammation, which may biologically pertain to stimuli that originates directly or indirectly (via pro-inflammatory cytokines and other substances released by cells in the innate immune system) from the source of ITMs. On the other hand, relying solely on local stimuli that originate from surrounding cells would be an insufficient deciding measure for neutrophils to pick a death pathway.

**Fig. 9:**
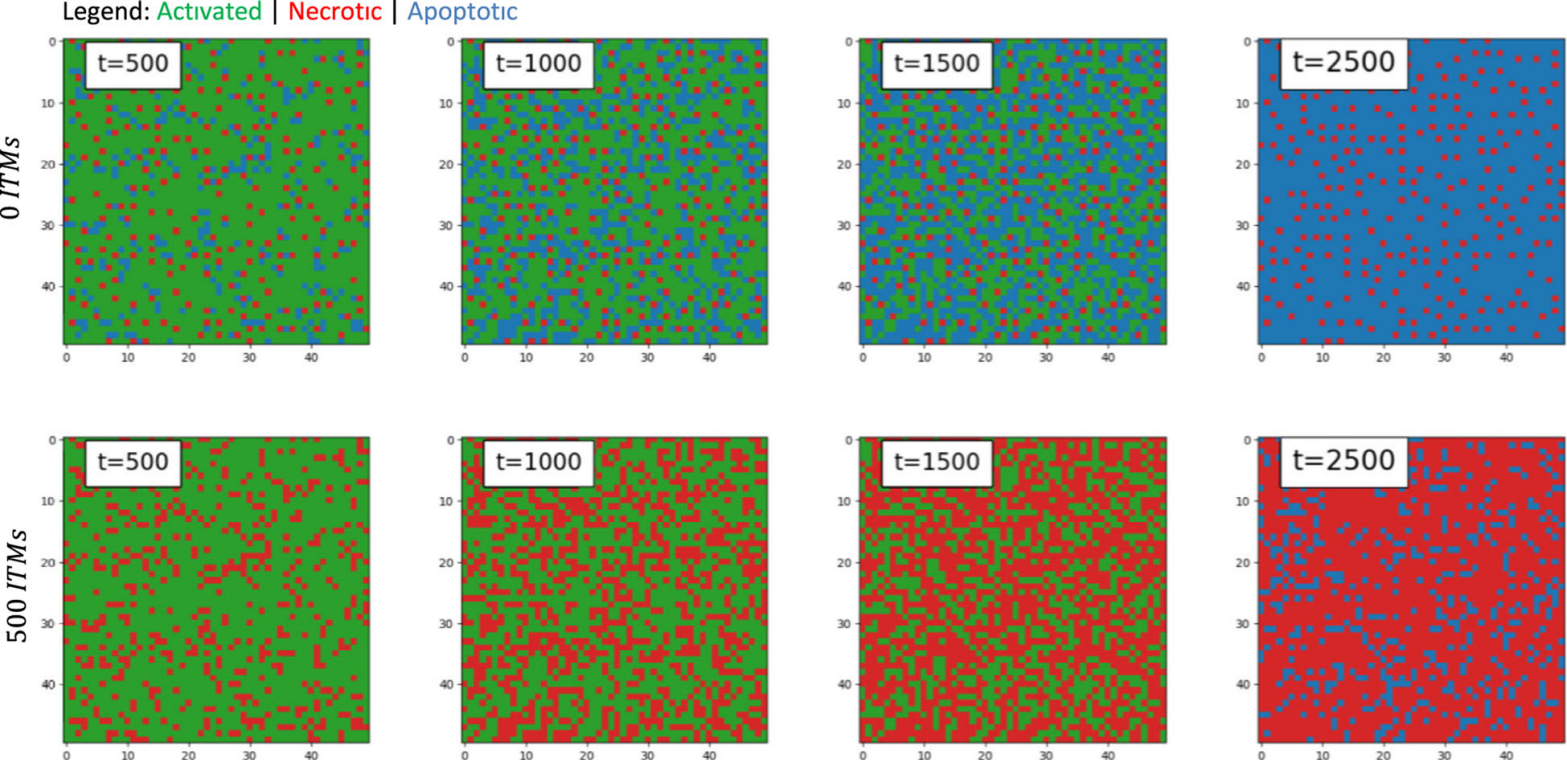
Global ITMs Scheme. Snapshots of the **50** ***×*** **50** lattice for 0 and 500 ITMs through iteration time (left to right). Green in the lattice corresponds to activated neutrophils, red to necrotic neutrophils, and blue to apoptotic neutrophils. Apoptosis (blue) remains dominant with less concentration of ITMs (top right). Conversely, necrosis (red) dominates with intensifying level of inflammation (bottom right). At 0 ITMs, necrotic neutrophils are better off isolated, which could be interpreted biologically as the system’s way to minimize local damage by allowing apoptotic neutrophils to settle in the space that separates them. Increasing the concentration of ITMs to 500, however reveals clustering of necrotic neutrophils together, which intensifies local tissue damage thus further aggravating the level of inflammation in the system

Snapshots of what the Global ITMs lattice looks like for various initial concentrations of ITMs evolving through time are summarized in Fig. 9. A single time step in our simulation corresponds to a single iteration in the algorithm where an activated neutrophil chooses a strategy based on a computed fitness. It is apparent that in a system with lower concentration of ITMs, necrotic neutrophils are better off isolated. This implies that their payoffs are maximized if they are situated away from the other necrotic entities, most likely to avoid aggravating the cost of necrosis which we interpret biologically as local tissue damage. On the other hand, apoptotic neutrophils tend to form clusters and position themselves in between necrotic neutrophils to maximize their payoffs. However, the tendency of necrotic neutrophils to isolate themselves is not evident in a system with higher concentration of ITMs. At 500 ITMs, we observe that necrotic neutrophils tend to cluster together, which can be biologically interpreted as the process when necrotic neutrophils intentionally aggravate inflammation by inducing local tissue damage.

We emphasize that the simulations shown in Fig. 9 aim to show how certain strategies dominate and propagate in the system through the course of time. Showing these dynamics provide a snapshot of how the system behaves at a particular point in time based on rules we specified in the payoff matrix. With minimal insult (0 ITMs), the system opts to take apoptosis as the optimal choice of strategy. However, when faced with an intense magnitude of insult (500 ITMs), activated neutrophils prefer to go into necrosis *early on* in the iteration, assumedly as an attempt to purposely aggravate the inflammation, which, biologically speaking, enhances the innate immune system’s response by recruiting more immune cells into the tissue. Apoptosis comes on later in the iteration as an attempt to exercise their anti-inflammatory benefits. Indeed, the higher the magnitude of insult is, the higher the *overall* benefit necrosis contributes to the survival of the system, which is not only prominent at the end point of the iteration, but also is observed at the beginning of the iteration process. In fact, this corresponds well to what is observed in data [9]. The authors were able to show that through the course of 36 h, apoptosis slowly increases and dominates when the insult is minimal. However, with higher magnitude of ITMs, apoptosis remains minimal, and only shows a slight increase at the end of the experiment (see Fig. 2 in [9]).

**Fig. 10:**
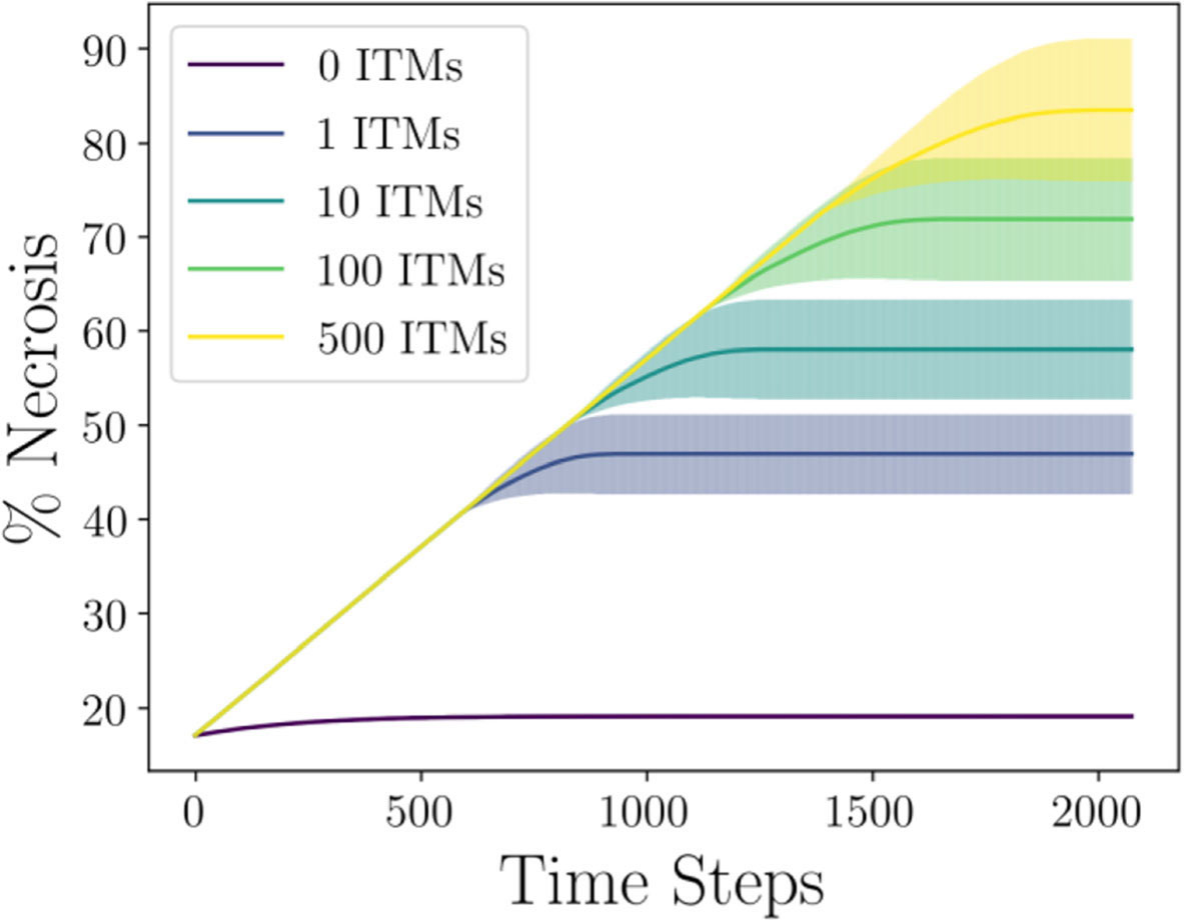
Cumulative number of necrotic neutrophils per time step in cellular automata. A single iteration corresponds to a single time step, where an activated neutrophil is made to choose a strategy. The plots correspond to the average number of necrotic neutrophils from 20 randomly chosen parameters in the data parameter space and the error bars correspond to the standard deviation

Using cellular automata to model the choice of death pathway reveals behaviors that emerge from microscopic interactions. We investigate these microscopic behaviors by looking at the cumulative number of activated neutrophils that go into necrosis per unit timestep. Our results are summarized in Fig. 10.

The bold lines in the plots correspond to the average number of necrotic neutrophils obtained per timestep by setting 20 combinations of parameters which we have randomly chosen from the data parameter space. Here we show that the cumulative number of activated neutrophils going into necrosis stabilizes at points that follow a linear trend with respect to the iteration time step. Note that a single time step corresponds to a single iteration where an activated neutrophil chooses to go into apoptosis or necrosis. This is simply because a single iteration limits a single activated neutrophil to go into apoptosis or necrosis. In our simulation, the preferred strategy in the early part of the iteration is necrosis, which means that pro-inflammatory functions due to local tissue damage comes first, followed by apoptosis, which induces anti-inflammatory signals. Note that each iteration step here does not directly correspond to the biological time frame. That is, we only look at the end point where stable strategies are achieved.

### What kind of game are the neutrophils playing?

In this section we identify where the neutrophil game lies among existing games known in game theory. Based on the payoff values specified in Table 1, it is easy to see that what we have is a symmetric game, where the payoffs are only dependent on the strategies employed, but not on the players. More so, the payoff inequality of *D* > *C* > *B* > *A* perfectly describes a so-called deadlock game, where the strategy that is mutually beneficial is also the most dominant – apoptosis. However, due to the added effect of global cost to the average payoff as in (5), instead of having a pure strategy dominating as the Nash equilibrium (apoptosis, in this case), a mixed Nash equilibrium is observed as stable equilibrium.

It is easy to show that a mixed Nash equilibrium does not exist when the global cost term is not taken into account. The average payoff *F*(*q*, *p*) of an individual playing necrotic with probability “ ” in a population, which overall plays necrotic with probability “ *p* ” would then be given by:

14 $$ F\left(q,p\right)= qpA+q\left(1-p\right)B+\left(1-q\right) pC+\left(1-q\right)\left(1-p\right)D $$

We can calculate the optimal *q* by taking the derivative of the average fitness cost as shown below:

15 $$ \frac{dF\left(q,p\right)}{\delta q}= pA+\left(1-p\right)B- pC-\left(1-p\right)D=0 $$

Substituting the exact values from the payoff matrix in Table 1 leads us to the following inequality:

$$ -p{c}_{Necrosis}+\left(1-p\right)\left(-{c}_{Necrosis}+{b}_{Apoptosis}\right)-p{b}_{Apoptosis}-\left(1-p\right)2{b}_{Apoptosis}=0 $$

16 $$ -{c}_{Necrosis}-{b}_{Apoptosis}\ne 0 $$

The value *p* is undefined when searching for a mixed equilibrium, where the fitness of going into apoptosis and that of necrosis is equal. This shows that indeed, a mixed Nash equilibrium does not exist for the game of neutrophils when the global cost term is omitted from the average fitness equation. This also implies that the global cost is necessary to replicate the percentage of neutrophils observed in data. This exact mechanism also matches with the cellular automata scheme, where we show that information on the *global* concentration of remaining ITMs in the body via direct or indirect stimuli is necessary in order for the neutrophils to function as they do to resolve inflammation in the body. This global rule that appears to be the driving force for cooperation, which we refer to as *necrosis*, has been explored in a previous study [32]. Indeed, having global information of the system is sufficient to drive the percentages of necrotic and apoptotic population that are needed to resolve the inflammation in the body.

## Summary and conclusion

Inspired by evolutionary game theory, we construct a game that describes the choice between two death pathways for neutrophils exposed to various levels of insult. Although the game of neutrophils resembles a dead lock game, the characteristics of this evolutionary game are those of a mixed strategy game where the evolutionary stable strategies are a combination of apoptotic and necrotic strategies. We demonstrate that using the payoff matrix alone cannot describe the stable evolutionary states that lead to the delicate balance between the two death pathways. Instead, the global cost of remaining ITMs is necessary to replicate the percentage of neutrophils observed in data. Using our model, we reproduce the power-law behavior exhibited by the percentage of necrotic neutrophils with respect to different levels of ITMs in data. We also use the data to reconstruct the space of possible evolutionary games which nature could have played to optimize the balance between apoptosis and necrosis depending on the level of insult. Using evolutionary game theory, we are able to identify and relate the mechanisms such as the benefit of apoptosis, cost of necrosis, strength of ITM resolution by apoptotic and necrotic neutrophils, and the global cost factor of remaining ITMs in the system that altogether contribute to the overall cost and benefit that establishes the optimal balance between necrosis.

Hence, using our simple model, we are able to pinpoint the driving mechanism that leads to the percentage of necrosis and apoptosis that reproduces the data – *global cost of remaining ITMs*. More importantly, by utilizing cellular automata, we provide numerical evidence that neutrophils need sufficient information of the scale of inflammation in order to choose a death pathway effectively.

## Data Availability

The data supporting the results of this research paper are included within this article. The model code is available via https://github.com/avpresbitero/GON and three-dimensional interactive plots shown in Figs. 5, 6 and 7 can be viewed online via https://github.com/avpresbitero/EGTN
